# A Multidisciplinary Approach to the Treatment of Traumatically Intruded Immature Incisors. A 6-Year Follow Up

**Published:** 2007-01-20

**Authors:** Mojtaba Vahid Golpayegani, Nikoo Tadayon

**Affiliations:** 1 *Department of Pedodontics, Dental School, Shahid Beheshti University of Medical Sciences, Tehran, Iran*; 2 *Department of Pediatric Dentistry, Shahid Beheshti University of Medical Sciences, Tehran, Iran*

**Keywords:** Force Eruption, Intrusion, Orthodontic Repositioning, Trauma

## Abstract

This report presents a case of 10 years old girl who was referred to the pediatric dentistry clinic sustaining a sever trauma led to crown fracture and intrusive luxation of immature maxillary incisors. Antibiotic therapies were initiated at first visit, and after surgical exposure both intruded and extruded teeth were endodontically treated by calcium hydroxide. Orthodontic repositioning was performed and root canal filling with gutta-percha was accomplished. Six years after orthodontic repositioning, clinical and radiographical examinations revealed satisfactory apical and periodontal conditions.

## INTRODUCTION

By definition, intrusion is an axial displacement of tooth into alveolar socket. Based on the pathogenesis of this type of injury, the impact has to come in an axial direction (1). If the force is in an axial-labial or an axial-lingual direction, the tooth may be displaced in apical-labial or apical-lingual directions. The former displacement results in a labial bone plate fracture (2).

In the established dentition, diagnosis is based on variation related to the position of the incisal edges of affected and unaffected teeth, whereas in the mixed dentition a high metallic note on percussion is indicative of intrusion or lateral luxation (1,3). The majorities of intruded teeth _because of their locked position in the socket_ are not sensitive to percussion and are completely firm (1). Due to the direction of dislocation, there is no occlusal disturbance and spontaneous pain is rare (4).

Radiographic examination reveals different apical levels, alveolar fractures or presence of damage to adjacent teeth (3). Sometimes a missing or diminished periodontal space can be seen in radiographs (1).

According to different studies, intrusion of permanent teeth is a rare injury with frequency ranging from 0.23 to 5 percent (2,5,6). The 6-12 years old age is the most frequently involved group in intrusion injuries, but it may also occurs later in life. Boys appear to suffer from intrusion earlier than girls. There appears to be a slight difference in the sex proportion of intrusion with more frequently exposure to intrusion in males. The maxillary central and lateral incisors are constitutes for the majority of cases while mandibular teeth are seldom involved (2).

The etiology of intrusions apparently differs in different countries; thus in Austria waterslides is the dominating cause (7), whereas in Denmark, common falls were the dominant etiology followed by bicycle injuries (2).

This paper describes the treatment of severely intruded and extruded maxillary central and lateral incisors in a 10 years old girl.

## CASE REPORT

A 10-years-old girl was brought to the private pediatric dental clinic two days after she had received trauma to her jaw and face due to car accident. She did not have any medical problem. Contusion of chin and lips could be observed in extra oral examination. Intra oral examination revealed gingival inflammation and bleeding from gingival sulcus of the incisors. Further examination revealed 1.5mm extrusion and complicated crown fracture of the maxillary left permanent central incisor, absence of the maxillary right permanent central incisor, 3mm intrusion and uncomplicated crown fracture of the maxillary right permanent lateral incisor. Subluxation of maxillary left lateral incisor was also noted.

**Figure 1:**
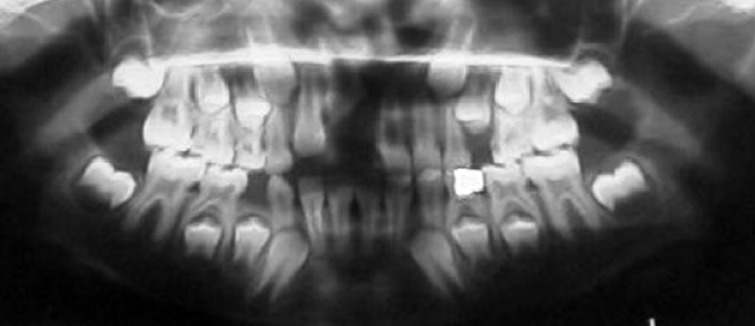
Panoramic radiography shows position of both intruded and extruded centrals in the maxilla

**Figure 2:**
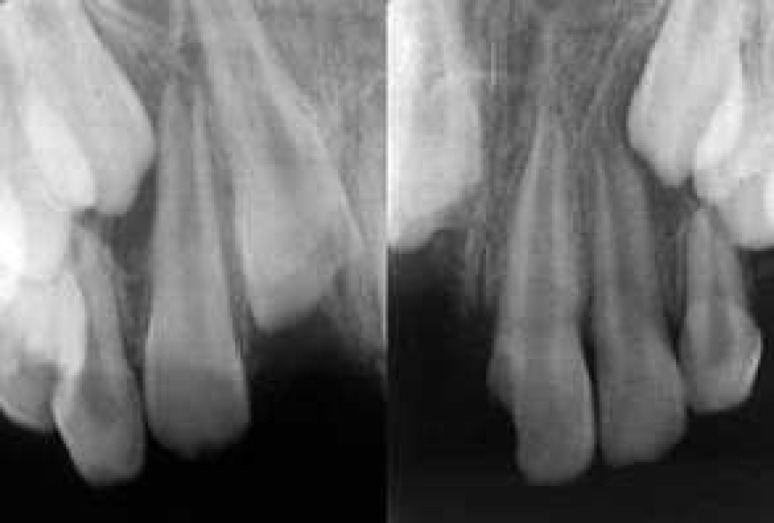
Periapical radiographies shows complete intrusion of right maxillary incisor

**Figure 3:**
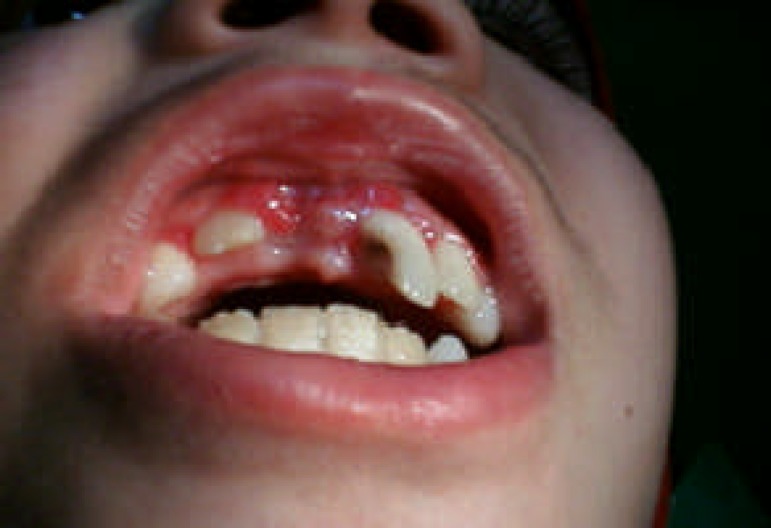
Clinical appearance 10 days after injury

The radiographic examination revealed complete intrusion of the maxillary right permanent central incisor, with concomitant crown fracture and pulp involvement (Figure 1 and Figure 2). Antibiotic was prescribed (Penicillin V, 500 mg, qid) for 5 days. Analgesics and mouth rinse (0.2% chlorhexidine gluconate solution) were also recommended. She was instructed not to bite with her anterior teeth and was encouraged to maintain oral hygiene. Her follow-up visits were also scheduled. After 10 days, the swelling had been subsided (Figure 3).

**Figure 4:**
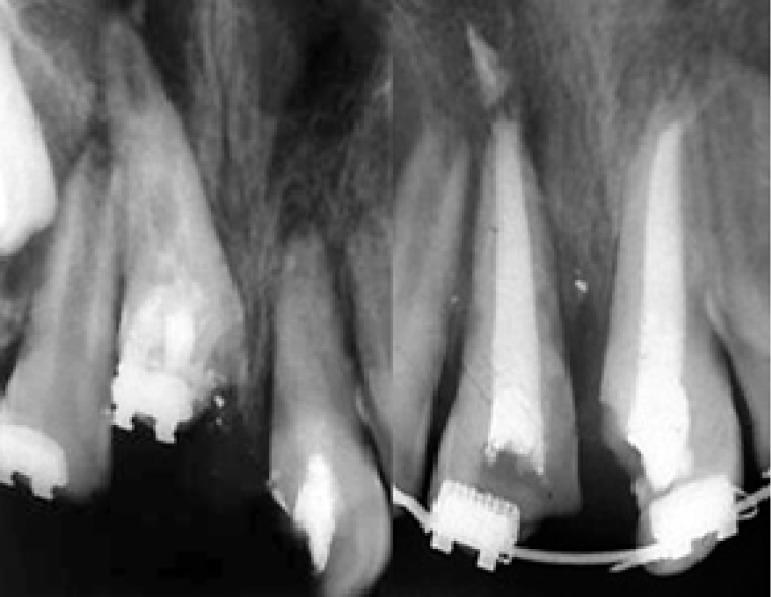
Intracanal medication and final RCT

Due to inaccessibility to the root canal of the maxillary right central incisor, surgical exposure was performed. The root canals of both central incisors were instrumented and filled with calcium hydroxide. The access cavity was sealed with reinforced zinc oxide eugenol cement and bracket was bonded to the labial surface of the intruded central (Figure 4).

**Figure 5:**
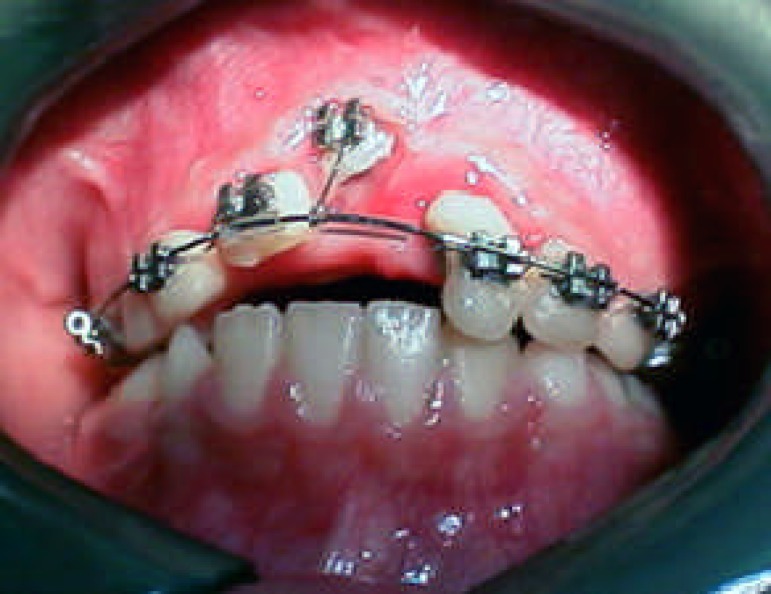
Intruded tooth is ligated to arch wire

At next visit, banding of maxillary second primary molars and bonding of permanent incisors and primary canines were performed. Round arch wire 0.018 was placed. The intruded central was ligated to the arch wire in order to help it’s eruption in an accurate axial direction. The maxillary left lateral was left free for spontaneous re-eruption (Figure 5).

**Figure 6:**
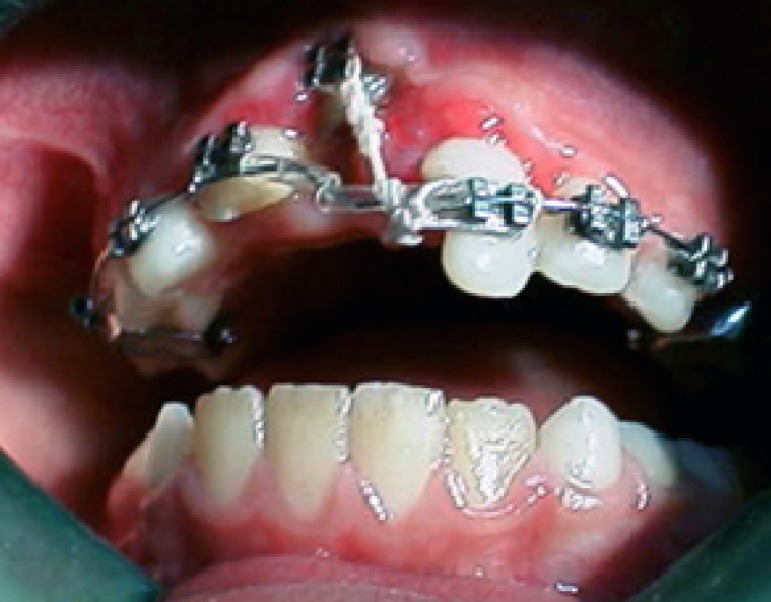
Ligature wire has been substituted by thread elastic

**Figure 7:**
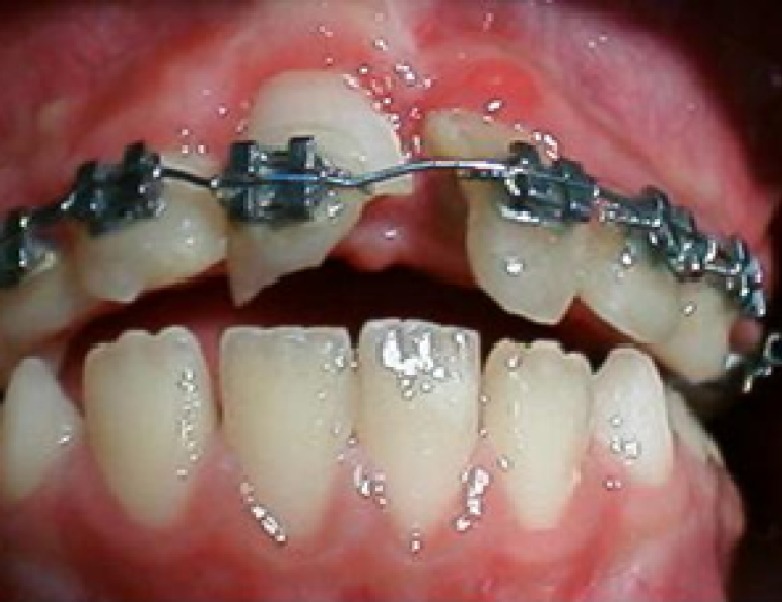
The intruded tooth in normal position

The root canal medication was changed twice more until the apical closure was confirmed radiographically. Two months after traumatic injury, clinical examination revealed that although the intruded incisor had moved to its half way, the procedure seemed to be very slow. It was decided to apply a lighter force on the intruded central by changing the ligature wire to thread elastic (Figure 6). After 4 months, the intruded tooth was replaced to its normal position. Further alignment was achieved by changing the position of the bracket on the central incisor and engagement of arch wire with the bracket of maxillary lateral incisor (Figure 7).

The radiograph revealed apical closure following 1 year treatment with calcium hydroxide. Both teeth were then filled with gutta-percha and root canal sealer (Figure 4). Crowns of both centrals were restored with a light-cured resin composite (Z100, 3M, USA).

The patient was scheduled for every 6-month follow ups.

At 6-month recall, the incisal level of left central incisor was appeared to be slightly infra occlusion in relation to the homologue, which gave rise to suspicion on ankylosis. A bracket was bonded to the labial surface of the same tooth and a Hawlly appliance was fabricated. She was instructed to wear the appliance for one year in order to level the right central with adjacent teeth and further stabilization in alveolar bone.

**Figure 8:**
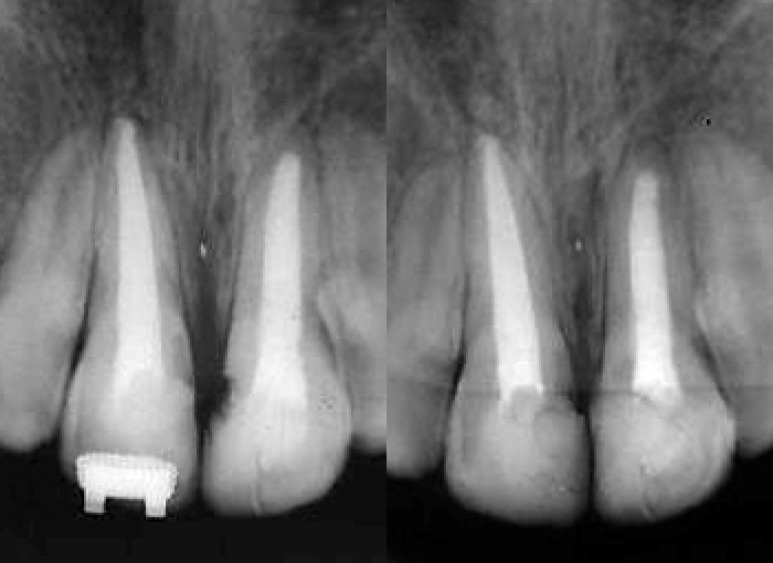
Radiographies at the end of the retention course (left) and 6-years follow up (right

**Figure 9:**
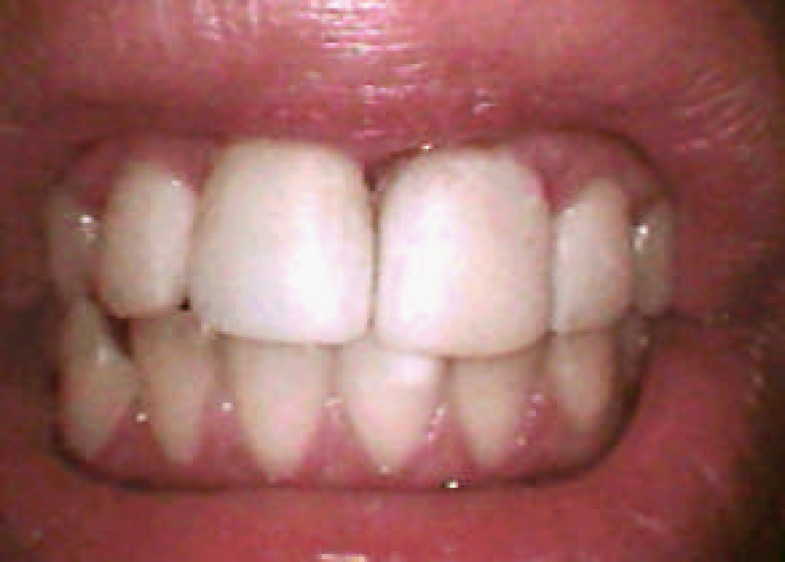
Clinical appearance at 6-year follow up

As shown in Figure 8 and Figure 9, at 6-years follow up both teeth were clinically asymptomatic and in function with healthy surrounding periodontal tissues. No loss of marginal bone support, pathologic mobility, periapical tenderness was observed. In the radiographic examination, however, healing without signs of external root resorption or periapical lesion was evident.

## DISCUSSION

One of the most damaging injuries to a tooth and its supporting structures is an intrusion luxation. These injuries are often accompanied by comminution or fracture of the alveolar socket (1,8). The low incidence of intrusion injuries may explain the paucity of knowledge about treatment of these cases (1). Decisions regarding the treatment vary according to the severity of intrusion and whether the tooth has a complete or incomplete root formation (3). The recommended treatment option includes:

1- Allowing spontaneous re-eruption 

2- Surgical repositioning

3- Orthodontic extrusion

The UK national clinical guidelines, related to this issue, suggest spontaneous re-eruption for the management of mildly intruded teeth (<3mm) with incomplete apex. The relative merits of orthodontically repositioning or spontaneous re-eruption for mildly intruded teeth with complete apex is unproven and treatment choice is by personal preference. Moderately intruded teeth (3-6mm) with incomplete apex can be repositioned either by orthodontic force or spontaneous re-eruption. Treatment choice is by clinical judgment and preference. Moderately intruded teeth with complete apex should be repositioned orthodontically. In the cases of severely intruded teeth (>6mm) with incomplete apex, the alveolus is grossly dilated labially and occasionally is fractured. There is often sever soft tissue displacement and the crown may be completely buried. Consideration should be given to surgically repositioning of tooth.

Severely intruded teeth with complete apex need to be repositioned surgically and appropriate tissue repair should be carried out. The difficulty in orthodontic repositioning of severely intruded teeth is the reason of suggestion for surgical repositioning (3).

The general outcome of intrusion with respect to both pulpal and periodontal ligament healing is not good. In fact, this trauma entity has the most serious prognosis among injuries affecting the permanent dentition (1).

It is not yet evident that which approach is most reliable. In any case, root canal treatment is a must (except in very immature teeth) and ankylosis-related resorption is a frequent occurrence. The only exception to root canal treatment is for the active repositioning in the very young, developing teeth (probably only below the age of 8 or 9) (9,10). Ankylosis is one of the main concerns during the course of orthodontic repositioning. For stability of anchorage, it is preferred to use rectangular arch wire and in case of round wire, a heavier one is recommended (9,10). In this case, round wire (0.018) was selected and at the half way of re-eruption, the ligature wire substitute by thread elastic in order to apply light pressure and prevent ankylosis. In teeth with immature root formation, apexification is initiated before definitive root filling with gutta-percha (1).

When minor fractures of the alveolar process is associated with traumatized teeth, bony fragments should be repositioned and soft tissue wounds sutured as necessary (11). Radiographs should be taken at each recall appointment to check the signs of apical periodontitis and progressive external resorption. Delay in examination and repositioning appeared not to be a significant factor but apparently reflected the severity of the injury. This implies that intruded permanent teeth generally do not demand an acute treatment approach (12).

The value of repositioning from a theoretical point of view may relieve compression zones in the periodontal and pulpal area and thereby facilitate healing. Secondly, the creation of the distance between root surface and the contused bone socket may favor cemental healing instead of ankylosis (13).

Antibiotic treatment is normally administered, usually penicillin in doses of 1000 mg immediately followed by 500 mg qid for 4 days (2). According to the UK national clinical guidelines the benefit of antibiotic treatment is unproven. Contusion of PDL and pulpal ischemia, are two factors that are not likely to be influenced by antibiotics (12).

A detailed analysis regarding the number of intruded teeth showed that when adjacent teeth were intruded, there was usually poor bone healing between the intruded teeth, while bony healing between the intruded teeth and adjacent non-injured teeth showed better healing. This phenomenon is possibly related to bone induction capacity of vital PDL of adjacent teeth. This is the finding of clinical and experimental assessment the both (14).

## CONCLUSION

The outcomes of this case report showed that the surgical exposure and endodontic treatment followed by orthodontic repositioning associated with long term retention is an alternative treatment for sever intrusive luxation in immature permanent teeth.
